# Perforated Caecum in a Left-sided Amyand’s Hernia: A Case Report

**DOI:** 10.31729/jnma.8114

**Published:** 2023-04-30

**Authors:** Saurav Jha, Arjun Kandel, Barsha Baral, Pradeep Ghimire

**Affiliations:** 1 Department of Emergency medicine, Manipal College of Medical Sciences, Phulbari, Pokhara, Nepal; 2 Nepal Medicity Hospital, Nakhkhu Ukalo Road, Kathmandu, Nepal; 3 Metrocity Hospital, Srijanachowk, Pokhara, Nepal; 4 Department of Surgery, Manipal College of Medical Sciences, Phulbari, Pokhara, Nepal

**Keywords:** *appendix*, *case reports*, *hernia*

## Abstract

Amyand's hernia is a rare type of inguinal hernia with an appendix inside an inguinal hernia sac. Most cases are diagnosed intraoperatively during hernia repair. A 66-year-old male was received at the Emergency Department with complaints of acute onset abdominal pain, vomiting, and groin swelling. The patient was diagnosed with obstructed left inguinoscrotal hernia with suspected bowel perforation. Following the emergency laparotomy, the intraoperative picture depicted a leftsided Amyand's hernia with a perforated caecum in the hernia sac. Mobile caecum, malrotation, situs inversus, and excessively long appendix denoted it to be the prime factors for the left-sided Amyand's hernia. A diverse range of pathological features and presentations might complicate the diagnosis and management of Amyand's hernia and all in all treatment has to be individualized according to the intraoperative finding.

## INTRODUCTION

Hernia containing vermiform appendix clarifies the diagnosis of Amyand's hernia.^1^ Incidence of Amyand's hernia is rare with a reported incidence between 0.1 to 0.4%. Most cases of Amyand's hernia are right-sided pertaining to the normal position of the appendix.^2^ Out of the handful of literature on left Amyand's hernia that is seldom encountered most of them are diagnosed intraoperatively during the management of incarcerated inguinal hernia and management of the case is usually determined by the pathology encountered. Preoperative diagnosis requires a high degree of clinical suspicion and imaging is the key. We, herein report a rare case of left-sided Amyand's hernia containing perforated caecum which is among a few scarce events ever encountered intraoperatively.

## CASE REPORT

**Figure 1:**
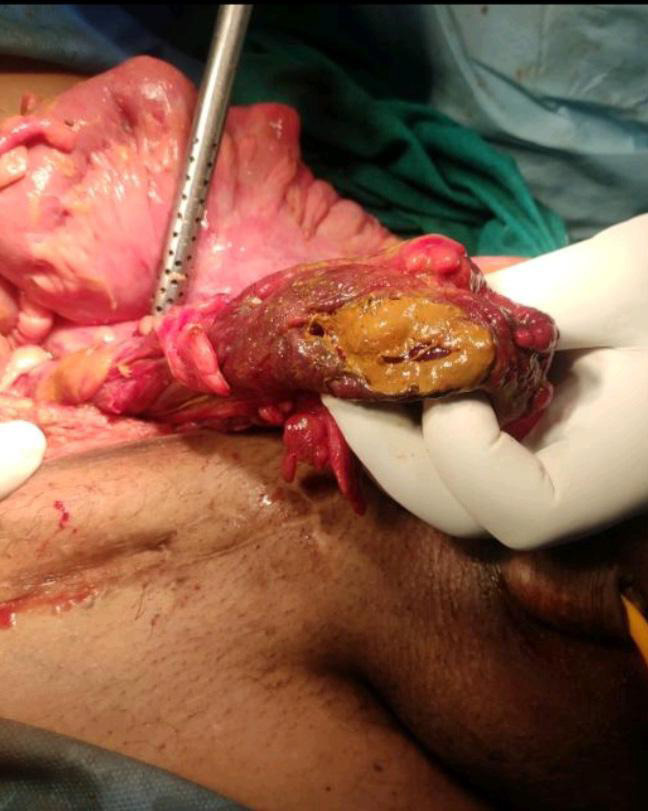
Intraoperative picture showing perforated caecum.

A 66-year-old male with a long-standing left-sided inguinal hernia was referred to our emergency with chief complaints of irreducible inguinoscrotal swelling for one day and increased pain abdomen since three hours. The pain was acute in onset, present in the left inguinoscrotal area, and dull aching associated with a few episodes of vomiting. On clinical examination he was vitally stable except blood pressure was 90/50 mm Hg in supine position on both arm and signs of dehydration was positive. Abdominal examination revealed a rigid abdomen with no bowel sound on auscultation. Local examination suggested a large irreducible left inguinoscrotal swelling reaching up to the base of the scrotum. Right-sided inguinoscrotal region seemed intact. Routine lab parameter was within normal limit except for raised serum urea and creatinine value. Lab investigation revealed the value of serum urea and serum creatinine as 95 mg/ dl and 4.5 mg/dl respectively. Abdominal X-ray was done and revealed gas under the diaphragm. Scrotal ultrasound depicted obstructed left inguinoscrotal hernia with dilated and aperistaltic bowel loop with minimum vascularity. At this stage, a provisional diagnosis of obstructed left inguinoscrotal hernia with perforated viscus and acute kidney injury was made. The patient was resuscitated with intravenous fluids, broad-spectrum antibiotics covering both anaerobic and aerobic organisms. The patient was then prepared for emergency laparotomy and proceeded with general anesthesia. Midline laparotomy incision was performed which was followed by the opening of the hernia sac. Appendix, omentum, and caecum were found during the surgery. Approximately, 4x4 cm irregular perforated margin was noted in the caecum along with an inguinal defect of 2 cm was visualized (Figure 1).

Fecal peritonitis was also observed which was then followed by aseptic peritoneal lavage and drainage. Contents were reduced and then the primary closure of caecal perforation and appendectomy along with herniorrhaphy was done. Temporary diversion ileostomy was created for the better healing of sutured perforated caecum. The postoperative period was uncertain with gradual decline in serum creatinine and urea level. Patient's condition symptomatically improved over subsequent days of hospital stay and patient was discharged on the 14^th^ postoperative day. Vitals were within normal limits with no fresh complaints and laparotomy wound was healthy at the time of discharge. Patient was discharged on antibiotics and advised to follow up after 6 months for closure of stoma. After 6-month stoma was closed and subsequently normal bowel function was regained with no significant complications.

## DISCUSSION

Hernia by definition is a protrusion of the viscus or part of the viscus through an abnormal opening in the wall of its containing cavity. Appendix, when present within hernia sac meets the criteria for Amyand's hernia. Claudius Amyand Surgeon at St Georges hospital performed the 1^st^ appendectomy for an inflamed appendix during a herniotomy of an 11-year child.^1^ Amyand's hernia is mostly diagnosed on the right side with reference to the normal anatomical position of appendix. Only 0.1% cases of right-sided Amyand's hernia have appendiceal inflammation present.^3,4^ There is a handful of literature describing the leftsided Amyand's hernia.^5^ Our patient had left-sided Amyand's hernia with a perforated caecum which is an extremely rare encounter.

Due to the relative paucity of cases and clinical presentation which is synonymous with incarcerated inguinal hernia and lack of adequate available literature exact pathophysiology, diagnostic criteria and management of Amyand's hernia is still lacking. Situs inversus, mobile caecum, mal-rotation and excessively long appendix are some of the theories purpose biased to the left sided Amyand's hernia.^5^ Mobile caecum was the most common clinical reason left behind Amyand's hernia in our patient. Appendix when found to be inflamed is usually due to adhesion causing compression of the appendix in the external ring. Obstruction caused by fecolith and lymphoid hyperplasia can also cause appendiceal inflammation in Amyand's hernia.

Presentation of Amyand's hernia varies with some cases being asymptomatic while most presenting clinically as an obstructed or strangulated inguinal hernia.^6^ A great number of cases of Amyand's hernia present in the form of incarcerated inguinal hernia and for such cases the usual standard treatment modality is emergent surgical repair so most of them are diagnosed intraoperatively with only a few asymptomatic cases diagnosed preoperatively via diagnostic radio imaging such as CT scan.^7,8^

Our patient presented with clinical features similar to strangulated inguinal hernia and rigidity of abdomen led to suspicion of perforated bowel content, henceforth management of the case proceeded accordingly. As in most cases, the diagnosis of Amyand's hernia containing perforated caecum was made intraoperatively. Usually, the management of Amyand's hernia is based on the contents, presence of appendix, and peritoneal inflammation. The appendix, when found to be inflamed, is to be removed and the mesh is avoided in case of peritonitis.^3,4^ Most surgeons wouldn't recommend removing appendix however some authors suggest removing appendix as it has a high chance of herniation and subsequent appendicitis.^3^ Losanoff and Basson have proposed a widely accepted initial tool for grading the management of Amyand's hernia into four categories.^9^ Losanoff and Basson grading proves to be a very useful guidance tool for the management of wide range of anomalies and pathology encountered in Amyand's hernia. However, in our patient, the intra-operative finding was caecal perforation with peritonitis and a non-inflamed appendix. So it categorically did not fall into any of the Losanoff types. Although the amount of literature pertaining to Amyand's hernia is found on increasing trend but to the author's knowledge this is among few rare cases of left Amyand's hernia with caecal perforation which was followed by caecal repair, appendectomy, and herniorrhaphy.

This case signifies how diverse variation is there in amyand's hernia and sometimes widely accepted tool for management of amyand's hernia might not be completely helpful in the proper treatment of individual variation encountered intraoperatively and the treatment has to be individualized for each patient. It also raises the awareness for surgeons to have high degree of suspicion for potential involvement of caecum. Limitations associated with this case was that the management protocol followed in our patient might not be same in other cases and the treatment guideline followed here could not be generalized for other individuals.
